# Case Report: Mycotic common carotid artery pseudoaneurysm in a child. A case report.

**DOI:** 10.12688/f1000research.54206.1

**Published:** 2021-07-14

**Authors:** Ben Mrad Imtinene, Rim Miri, Ben Mrad Melek, Wafa Aloui, Sobhi Mleyhi, Neila Ben Aba, Zairi Ihsen, Tawfik Kalfat, Raouf Denguir

**Affiliations:** 1 Cardiology department, Hbib Thameur Hospital, Tunis, University Tunis El Manar, TUNIS, 1068, Tunisia; 2 Cardiovascular surgery department, Rabta Hospital, Tunis, University Tunis El Manar, TUNIS, 1068, Tunisia; 3 Pediatric department, Mongi Slim Marsa Hospital, Tunis, University Tunis El Manar, TUNIS, 1068, Tunisia

**Keywords:** Mycotic, aneurysm, children, carotid

## Abstract

Extracranial carotid artery aneurysms in children are extremely rare, nevertheless associated with a great potential of thromboembolic episodes and rupture especially those with mycotic origin. The surgical treatment is very challenging, and there is still a controversy concerning revascularisation after the resection of the aneurysm.

In this manuscript, we report the observation of an 8-year-old boy with the medical history of Leukemia who is admitted urgently for a mycotic right common carotid artery aneurysm, occurring after a chemoport infection who was operated on in our cardiovascular surgery department with surgical resection and ligation. It is the second report in the pediatric literature of a mycotic pseudoaneurysm situated in the common carotid artery, but the first documented by medical imagery. Through this case, we highlight that ligation of the infected carotid artery can be a safe and efficient alternative especially in Children.

## Introduction

Mycotic extracranial carotid artery aneurysm (ECAA) in children is extremely rare but associated with a high potential for rupture and thromboembolic episodes. ^1^ The surgical management is challenging and subject to controversy concerning the adequate technique.

In this article, we present an 8-year-old boy with a case of mycotic ECAA who was operated on in our cardiovascular surgery department. It is the second report in the literature of a mycotic pseudoaneurysm located in the common carotid artery. ^2, 3^

## Case report

An eight-year-old male North African child treated for leukemia was referred to our cardiovascular surgery department for management of a right lateral-cervical mass. The patient did not report any history of tonsillectomy or cervical trauma. The child was being followed for acute lymphoblastic leukemia type B for which he was receiving polychemotherapy. He had central venous ports (chemoports) placed via the right internal jugular vein one month prior to admission. The chemoport was complicated by a venous thrombosis with port chamber infection two weeks after its implantation. The chamber and the central venous catheter were removed, and the child received a double intravenous broad-spectrum antibiotic (Amoxicillin-Clavulanic Acid and Sulfamethoxazole) treatment two weeks before admission. The patient subsequently developed a right latero-cervical mass one day before admission.

**Figure 1.**
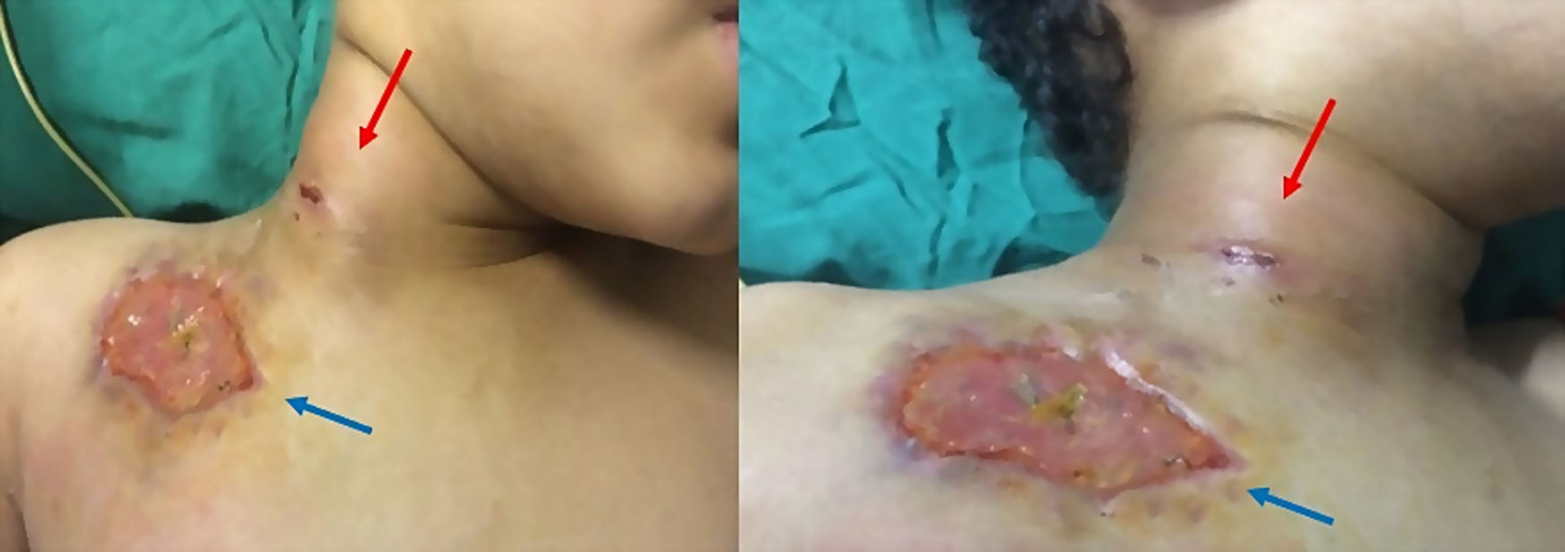
Right latero-cervical mass (3 cm of large axis), with inflammatory signs all around (red arrow) with cutaneous ulceration 5 cm in diameter located under the right clavicular (blue arrow) (former location of the implantable chamber).

Upon physical examination, the child was in good general condition, awake and alert. He was feverish (temperature = 38.9 EC); cardiac and pulmonary examinations were normal. Neurological examination was normal, notably without signs of facial weakness. Otorhinolaryngological examination was normal, ears and nose were clean. The pharynx exam was also normal, notably without erythema, exudates, or pharyngeal oedema. The patient had a right latero-cervical tumefaction ( Figure 1) painful to palpation, pulsatile and expansive (3 cm-long large axis), with inflammatory signs all around.

He also presented a cutaneous ulceration with a diameter of 5 cm, located under the right clavicle (former location of the implantable chamber), with inflammatory signs all around ( Figure 1). Complete blood count revealed anaemia with a haemoglobin level of 9.6 g/dL (Normal: 11.9-15 g/dl), thrombocytopenia with platelet levels of 126,000 per mcL (Normal: 150000-400000 mcL), and hyperleukocytosis with a leukocyte count of 22,3 × 10 ^9^/L (Normal: 4.5-14.5 × 10 ^9^/L). The C-reactive protein reached 95 mg/dl. (Normal: < 5 mg/dl).

**Figure 2.**
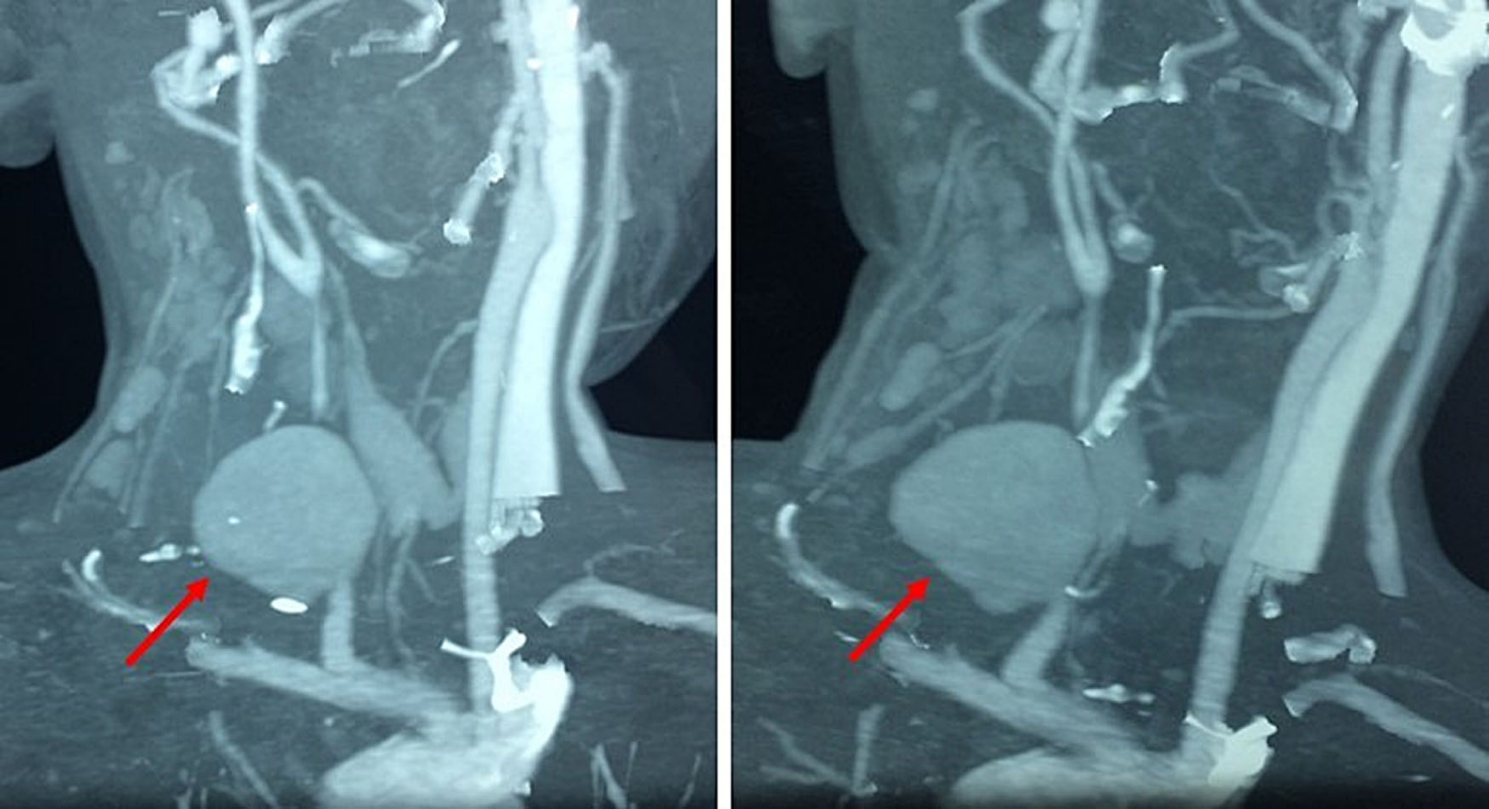
Cervical computed tomography angiogram showing a 3 cm right common carotid artery pseudoaneurysm beginning at 2 cm of its origin and at a distance from the carotid bifurcation (red arrow), with the presence of multiple cervical lymphadenopathies.

A cervical ultrasound found a right common carotid artery (CCA) false aneurysm measuring 29-28 mm of axis, with inflammation of the right cervical subcutaneous tissue associated with huge cervical lymphadenopathies. A cervical Angio-Computed Tomography (CT) scan ( Figure 2) showed a 3 cm pseudoaneurysm of the right CCA beginning 2 cm from its origin and at a distance from the carotid bifurcation, with the presence of multiple cervical lymphadenopathies and an unimpaired cerebral circulation through the polygon of Willis.

Haemocultures were carried out but did not find any isolated pathogens.

**Figure 3.**
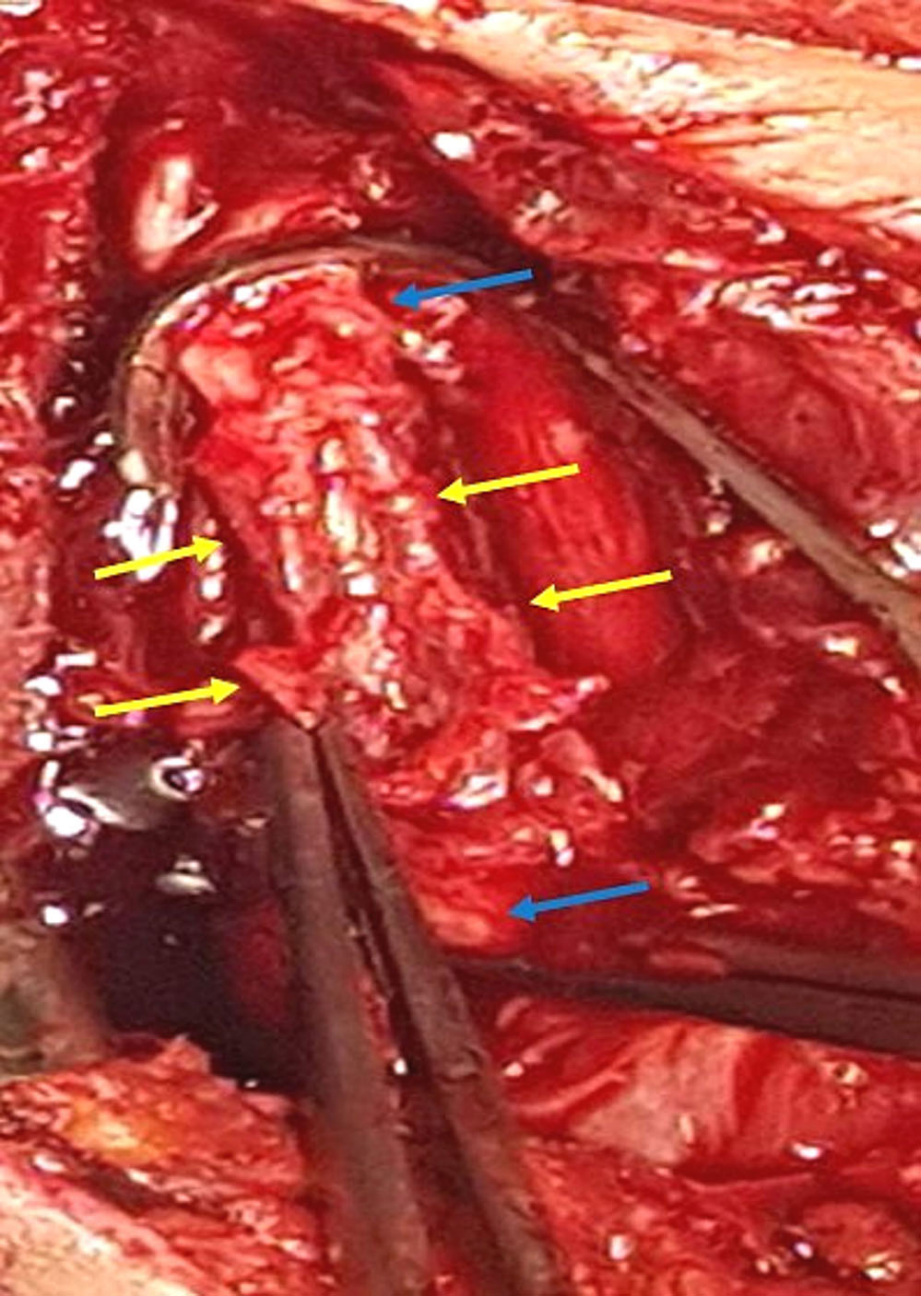
Preoperative view, the CCA was dissected and controlled and clamped at its origin and terminated just before the carotid bifurcation (blue arrow). Mycotic aneurysm, and all-around infected tissues were resected (yellow arrow).

**Figure 4.**
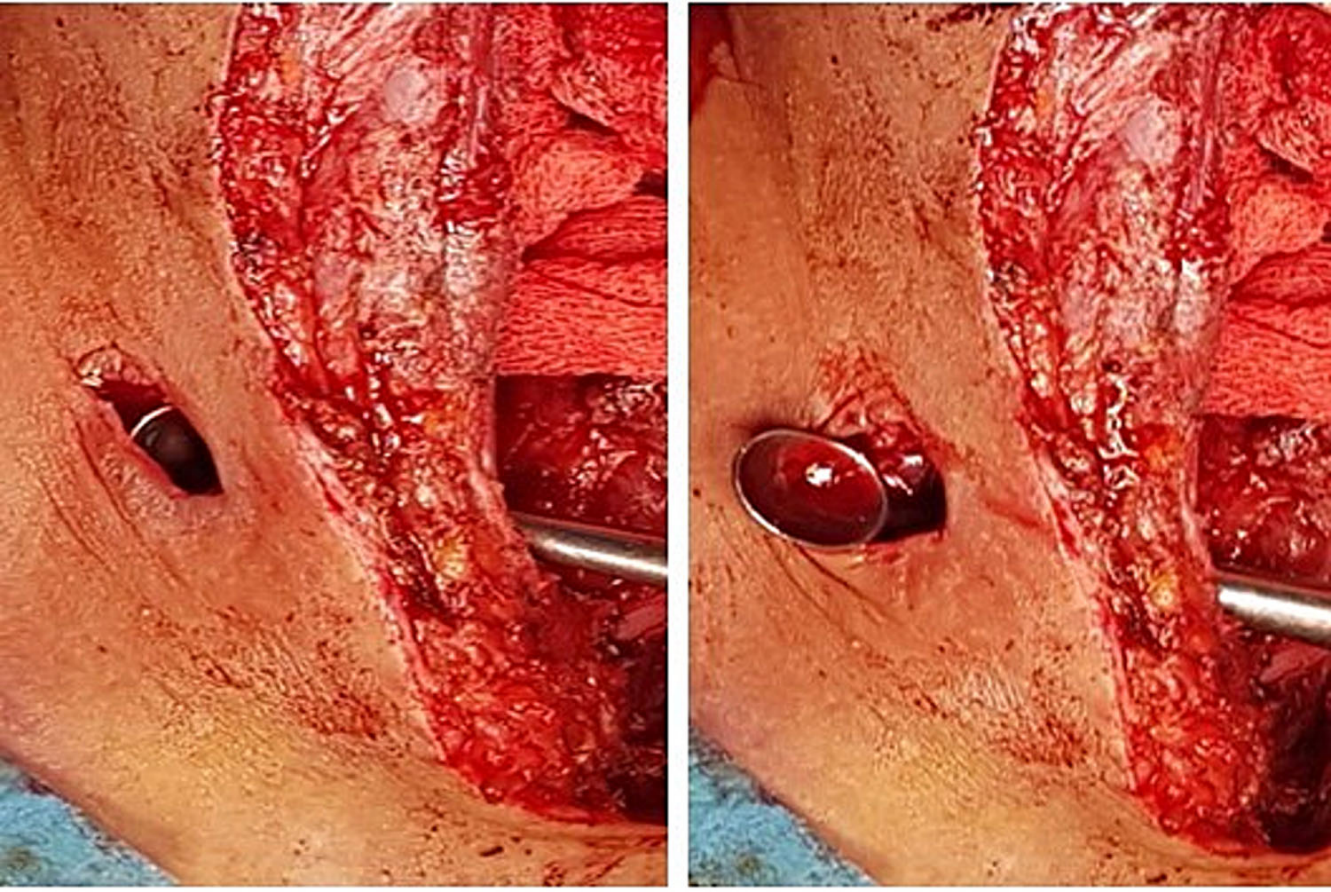
Preoperative view showing a cervical cutaneous fistula.

**Figure 5.**
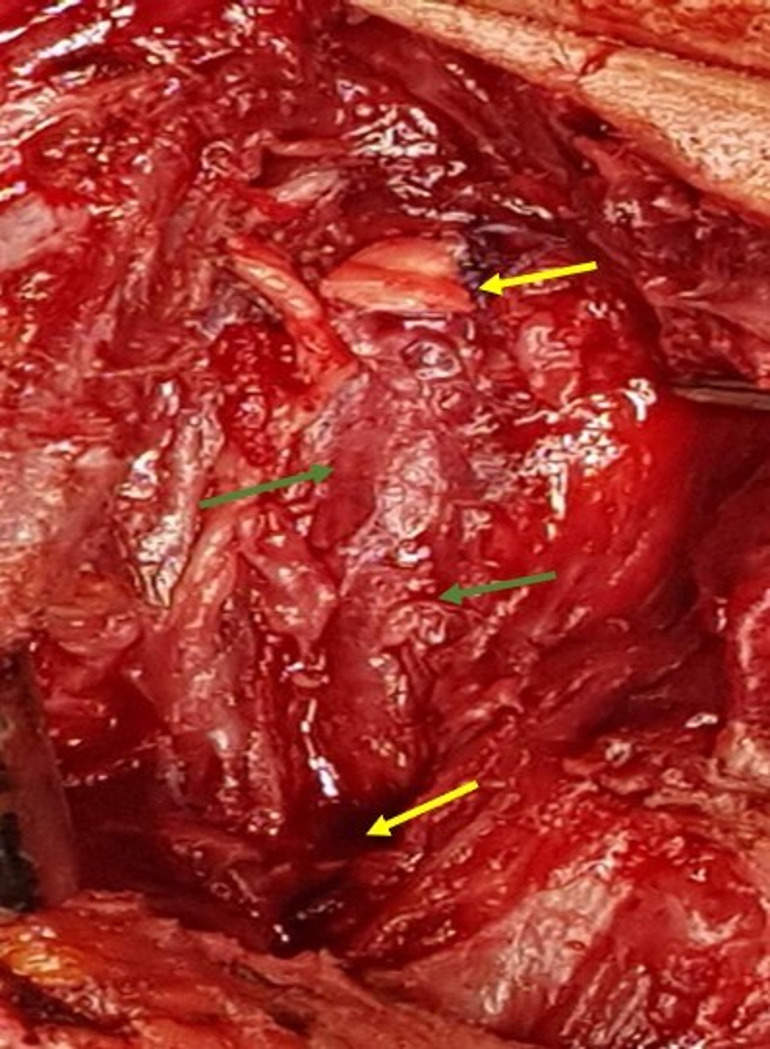
Preoperative view showing ligation of the two extremities of the CCA (yellow arrow) after total debridement of the infected area (green arrow).

The patient was operated on the evening of his admission. The procedure was carried out under general anaesthesia. The surgical incision was a lateral right cervicotomy extended to the sternal fork. First, the CCA was dissected and controlled at its origin and terminated just before the carotid bifurcation ( Figure 3). An arterial clamping was made on both sides of the mycotic aneurysm, and resection of the latter, and all-around infected tissues, was performed ( Figure 3). Our first strategy was to use the greater saphenous vein for reparation. However, given the extent of the infection with brittle arterial tissue, the existence of cutaneous fistula ( Figure 4), and the excellent pulsatile arterial reflux from the CCA, the decision was made to make a simple ligation of the artery to avoid complications, especially graft rupture ( Figure 5). The immediate surgical procedure was simple, with an alarm clock on the operating table and without neurological deficit.

During the postoperative period, the child was hospitalized in the intensive care unit. Excised local lymph nodes and aneurysm tissues were sent for bacteriological and pathologic examination. The culture of bacteriological samples was negative, a predictable result given the probabilistic antibiotic therapy introduced from the first presentation of symptoms. Repeated haematological analysis showed that his leukocyte levels had dropped to normal values.

The patient made an ordinary recovery and was discharged after two weeks. Low-dose aspirin (75 mg per day) with 6 weeks of oral antibiotics (Amoxicillin/Clavulanate 15 mg/kg every 12 hours) were prescribed. Upon one-year follow-up, the neurological examination was normal, and in particular showed no neurological sequelae.

## Discussion

Extracranial carotid aneurysms are uncommon in the paediatric population. Therefore, their natural history remains unclear. A literature review that was published in 2021 revealed that only 26 cases of infectious extracranial carotid artery pseudoaneurysms had been reported since 1990. ^3^

Most of the infectious extracranial carotid pseudoaneurysms occur in the internal carotid artery. ^1^ The first case of an extracranial pseudoaneurysm of the common carotid artery was reported by Willemsen in 1997; ^2^ our report is the second one in the medical literature.

In childhood, ECCA formation is mainly secondary to infection or trauma. ^4^ Historically, ear/nose/throat infections are associated with some vascular complications, such as the Lemierre syndrome (thrombosis of the internal jugular vein) and, uncommonly, carotid artery false aneurysm. ^5, 6^ However, these complications have become exceptional nowadays due to the widespread use of antibiotics. ^7^

The physiopathogenesis of the carotid false aneurysm in this case is not completely clear. We thought that the contiguous dissemination of infection into the parapharyngeal area appeared to be the main cause. In the study herein, the child had an infection of the chemoport implanted via the right internal jugular vein, which we presumed to be responsible for this deep neck infection. Pseudoaneurysm formation is secondary to fragilization and dilatation of the arterial wall by infectious arteritis. ^7^ This infection was promoted by the state of immunodepression caused by his leukemia. The interval separating the initial infection and the diagnosis of the pseudoaneurysm was 15 days in our case. This is in line with the data reported in the literature. ^7^

Pulsatile cervical mass is the most frequent clinical presentation of ECCA in the surgical literature. ^8^ Other clinical signs are dyspnea, dysphagia, and voice hoarseness by compression of adjacent anatomical and nervous structures. ^10- 12^ Neurological symptoms have been noted in relation with either a cerebral infraction or Horner’s syndrome. ^1^

In addition, a patient may present with a haemorrhage secondary to the rupture of the pseudoaneurysm. ^9^ This complication is more frequent with mycotic aneurysm. ^9^ Pourhassan *et al.*, reported a rupture rate of around 42% in their review of literature concerning carotid aneurysms in children. ^1^

The diagnosis in this case was based on cervical Doppler ultrasound and CT angiogram. ^5– 7^ It is highly necessary to assess the Willis polygon and the structures surrounding the aneurysm, before any possible surgical procedure in order to reduce complications. Cervical angiography is performed only if an endovascular treatment is considered. ^5, 7^

It is important to note the lack of evidence-based treatment guidelines for this complication in paediatric patients. However, given the high risk of rupture, urgent intervention is highly recommended. ^13^

Several treatment strategies with different levels of efficacy and limitations are available in cases of children with infectious extracranial carotid pseudoaneurysm, including surgical treatment, endovascular treatment, or a combination of the two. ^3^

Pseudoaneurysm resection with restoration of the arterial continuity using a saphenous venous graft is the most habitual surgical treatment. ^8^ However, in children, arterial reparation may not be realizable if the greater saphenous vein is small in diameter. In these cases, ligation can be proposed. In addition, reconstructive techniques are challenging because of inflammation and proximity of cranial nerves. ^13^

A few cases of ligation of CCA or ICA among the paediatric population, as in our case, have been reported in the literature. ^1^ The risk of stroke is relatively low in children in contrast with adults. ^6, 14^ However, we can only perform ligation if the collateral circulation is intact, in order to minimize neurologic consequences. ^6, 11, 15, 16^

Endovascular techniques such as stenting, and coil embolization have provided a less invasive approach to the treatment of infectious pseudoaneurysms in children. ^12^ However, there is still concern that coil embolization or stenting for infectious area may expose patients to an increased risk of persistent infection. ^12^ In all cases, we must combine a 4–6 weeks broad-spectrum antibiotic therapy with the chosen surgical or endovascular treatment. ^7^

Like our patient, in the majority of paediatric cases, an uneventful early outcome is reported. ^1^ However, there is a lack of data on long-term consequences.

## Conclusion

Mycotic carotid pseudoaneurysm is a rare complication in children, but associated with a high risk of fatal rupture. When diagnosed, it should be treated urgently. Surgical ligation seems to be a reasonable and viable choice of treatment, especially in children.

## Consent

Written informed consent for publication of the patient’s clinical details and/or clinical images was obtained from the father of the patient.

## Data Availability

All data underlying the results are available as part of the article and no additional source data are required.
