# Supplementary material for: Case Report: Mycotic common carotid artery pseudoaneurysm in a child. A case report
Source: F1000Res. 2021 Jul 14;10:564. [Version 1] doi: 10.12688/f1000research.54206.1 (PMC8567690; doi:10.12688/f1000research.54206.1)
Supplement: Peer review history

# Peer review: Reviewer response for version 1

**DOI:** 10.5256/f1000research.57667.r96569

**Published:** 2021-11-03

**Authors:** Maurizio Domanin

**Affiliations:** 1 Vascular Surgery Unit, Istituto di Ricovero e Cura a Carattere Scientifico Ca Granda Ospedale Maggiore Policlinico, Milan, Italy; 2 Dipartimento di Scienze Cliniche e di Comunità, Università degli Studi di Milano, Milan, Italy

The case presented here is well described by the authors, such as all the possible options for its repair have been deeply analyzed.

 In my personal experience, in particular in children or younger people, I always suggest exploring the possibility of arterial reconstruction. My personal experience on a similar case, but without any spread of local infection that could be eventually inserted among the references, was to reconstruct the whole carotid bifurcation using the greater saphenous vein and one of its major tributaries (Giacomini’s vein) (please see Domanin *et al*., 2018 ^1^). If feasible, biological grafts are resistant to infections and guarantee high rates of patency in the long term.

 Anyway, also the choice of the ligature of the carotid artery can be successful, if a valid collateral circulation, sustained by the contralateral carotid or by the vertebral circle, is present and well documented both by Doppler Ultrasound and Computed Tomography Angiography (or Magnetic Resonance Angiography). Moreover, a valid backflow, together with the measurements of the stump pressure from the ipsilateral internal carotid, can give further valuable information regarding the status of collateral supply to the brain.

 The background of the case’s history and progression is well detailed even if, in all these cases, the real event which leads to pseudoaneurysm development can only be supposed.

 Anyway, the work of Imtinene *et al.* is clearly presented with updates on the most recent literature regarding this niche topic and conclusions are correct and well balanced. The major problem is related to the extreme rarities of such cases and, as reported by the authors, the complete lack of evidence and the absence of guidelines.

 In this way, I think that every report on bailout treatments for arterial disease in newborns or children can be helpful for all the surgical community in order to provide and share therapeutic ideas for these really challenging cases.

 I congratulate the authors for their very interesting study which turns the lights on in this disputed topic.

Are enough details provided of any physical examination and diagnostic tests, treatment given and outcomes?

Yes

Is the case presented with sufficient detail to be useful for other practitioners?

Yes

Is sufficient discussion included of the importance of the findings and their relevance to future understanding of disease processes, diagnosis or treatment?

Yes

Is the background of the case’s history and progression described in sufficient detail?

Yes

Reviewer Expertise:

Vascular surgery

I confirm that I have read this submission and believe that I have an appropriate level of expertise to confirm that it is of an acceptable scientific standard.

## References

[1] : A Rare Cause of Juvenile Stroke: Extracranial Carotid Artery Aneurysm with Venous Complete Reconstruction of the Carotid Bifurcation. *Pediatr Neurosurg* .2018;53(4) : 10.1159/000487089 275-279 10.1159/000487089 29694968

# Peer review: Reviewer response for version 1

**DOI:** 10.5256/f1000research.57667.r96201

**Published:** 2021-10-27

**Authors:** Fabien Koskas

**Affiliations:** 1 Vascular Surgery Department, University Hospital La Pitié-Salpêtrière, Paris, France; 2 Paris Sorbonne Université, Paris, France

In this paper, an original pediatric case of mycotic aneurysm of the common carotid is presented and well documented. Given the seldomness of such cases, the case deserves publication. Strategy and tactics in emergency are well explained, justified and post-hoc-approved by the uneventful outcome. The paper will be particularly useful for a young vascular surgeon confronted with such an unusual challenge, especially in emergency. Moreover, the discussion on whether or not revascularize is particularly relevant from a medicolegal point of view. Young surgeons may be confronted with septic disasters affecting arteries of potentially vital arteries. There is then a choice between ligating to minimize the hemorrhagic risk but with the sacrifice of an artery of vital or functional importance or revascularizing, a much more complex procedure with an eventual risk of hemorrhagic, therefore vital complications. Strategic decisions must then be taken often in emergency and such decisions medicolegally expose the taker if eventually the vital of functional prognosis of the patient is affected. In our societies where the medicolegal issue is more and more a concern, it is important that published papers like this one show that the decision of ligating a potentially vital artery like the common carotid does not always result in a cerebrovascular disaster. I strongly recommend indexing.

Are enough details provided of any physical examination and diagnostic tests, treatment given and outcomes?

Yes

Is the case presented with sufficient detail to be useful for other practitioners?

Yes

Is sufficient discussion included of the importance of the findings and their relevance to future understanding of disease processes, diagnosis or treatment?

Yes

Is the background of the case’s history and progression described in sufficient detail?

Yes

Reviewer Expertise:

Vascular Surgery

I confirm that I have read this submission and believe that I have an appropriate level of expertise to confirm that it is of an acceptable scientific standard.
